# Diagnostics and correction of batch effects in large‐scale proteomic studies: a tutorial

**DOI:** 10.15252/msb.202110240

**Published:** 2021-08-25

**Authors:** Jelena Čuklina, Chloe H Lee, Evan G Williams, Tatjana Sajic, Ben C Collins, María Rodríguez Martínez, Varun S Sharma, Fabian Wendt, Sandra Goetze, Gregory R Keele, Bernd Wollscheid, Ruedi Aebersold, Patrick G A Pedrioli

**Affiliations:** ^1^ Department of Biology Institute of Molecular Systems Biology ETH Zurich Zurich Switzerland; ^2^ PhD Program in Systems Biology University of Zurich and ETH Zurich Zurich Switzerland; ^3^ IBM Research Europe Rüschlikon Switzerland; ^4^ Luxembourg Centre for Systems Biomedicine University of Luxembourg Luxembourg Luxembourg; ^5^ Queen’s University Belfast Belfast UK; ^6^ Department of Health Sciences and Technology Institute of Translational Medicine ETH Zurich Zurich Switzerland; ^7^ ETH Zürich PHRT‐CPAC Zürich Switzerland; ^8^ SIB Swiss Institute of Bioinformatics Lausanne Switzerland; ^9^ The Jackson Laboratory Bar Harbor ME USA; ^10^ Faculty of Science University of Zurich Zurich Switzerland

**Keywords:** batch effects, data analysis, large‐scale proteomics, normalization, quantitative proteomics, Proteomics

## Abstract

Advancements in mass spectrometry‐based proteomics have enabled experiments encompassing hundreds of samples. While these large sample sets deliver much‐needed statistical power, handling them introduces technical variability known as batch effects. Here, we present a step‐by‐step protocol for the assessment, normalization, and batch correction of proteomic data. We review established methodologies from related fields and describe solutions specific to proteomic challenges, such as ion intensity drift and missing values in quantitative feature matrices. Finally, we compile a set of techniques that enable control of batch effect adjustment quality. We provide an R package, "proBatch", containing functions required for each step of the protocol. We demonstrate the utility of this methodology on five proteomic datasets each encompassing hundreds of samples and consisting of multiple experimental designs. In conclusion, we provide guidelines and tools to make the extraction of true biological signal from large proteomic studies more robust and transparent, ultimately facilitating reliable and reproducible research in clinical proteomics and systems biology.

## Graphical abstract

In mass spectrometry‐based proteomics, handling large sample sets introduces technical variability known as batch effects. This tutorial provides guidelines and tools for the assessment, normalization, and batch correction of proteomics data.

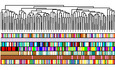

## Introduction

Recent advances in mass spectrometry (MS)‐based proteomic approaches have significantly increased sample throughput and quantitative reproducibility. As a consequence, large‐scale studies consisting of hundreds of samples are becoming increasingly common (Zhang *et al*, 2014, 2016; Liu *et al*, 2015; Mertins *et al*, 2016; Okada *et al*, 2016; Williams *et al*, 2016; Collins *et al*, 2017; Sajic *et al*, 2018). These technological and methodological advances, combined with proteins being the main regulators of the majority of biological processes, make MS‐based proteomics a key methodology for studying physiological processes and diseases (Schubert *et al*, 2017). MS‐derived quantitative measurements on thousands of proteins can, however, be affected by differences in sample preparation and data acquisition conditions such as different technicians, reagent batches, or changes in instrumentation. This phenomenon, known as “batch effects”, introduces noise that reduces the statistical power to detect the true biological signal. In the most severe cases, the biological signal ends up correlating with technical variables, leading to concerns about the validity of the biological conclusions (Petricoin *et al*, 2002; Hu *et al*, 2005; Akey *et al*, 2007; Leek *et al*, 2010).

Batch effects have been extensively discussed, both in the genomic community that made major contributions to the problem about a decade ago (Leek *et al*, 2010; Luo *et al*, 2010; Chen *et al*, 2011; Dillies *et al*, 2013; Lazar *et al*, 2013; Chawade *et al*, 2014) and in the proteomic community which has faced the issue quite recently (Gregori *et al*, 2012; Karpievitch *et al*, 2012; Chawade *et al*, 2014; Välikangas *et al*, 2018). Nevertheless, finding solutions to the problem of batch effects is still a topic of active research. Although extensive reviews have been written on the topic (Leek *et al*, 2010; Lazar *et al*, 2013), researchers still get confused about the terminology. For example, the distinction between normalization, batch effect correction, and batch effect adjustments is not always clear and these terms are often used interchangeably. To clarify how we use these terms in this Review, we compiled a glossary, found in Table 1. Some definitions are adapted from Leek *et al*, 2010.

**Table 1:** Terminology.

Term	Definition
Batch effects	Systematic differences between the measurements due to technical factors, such as sample or reagent batches.
Normalization	Sample‐wide adjustment of the data with the intention to bring the distribution of measured quantities into alignment. Most prominently, sample means and medians are aligned after normalization.
Batch effect correction	Data transformation procedure that corrects quantities of specific features (genes, peptides, metabolites) across samples, to reduce differences that are associated with technical factors, recorded in the experimental protocol (i.e., sample preparation or measurement batches). Usually samples are assumed to be normalized prior to batch effect correction. This step is often called "batch effect removal" or "batch effect adjustment" in the literature. Note the difference in the definition used here.
Batch effect adjustment	Data transformation procedure that adjusts for differences between samples due to technical factors that altered the data (sample‐wise and/or feature‐wise). The fundamental objective of the batch effect adjustment is to make all samples comparable for a meaningful biological analysis. In our definition, batch effect adjustment is a two‐step transformation: first normalization, then batch effect correction. Performing normalization first helps feature‐level batch effect correction by first alleviating sample level discrepancies.

There is also considerable debate on which batch correction method performs best, and multiple articles have compared various methods (Luo *et al*, 2010; Chen *et al*, 2011; Chawade *et al*, 2014). Other publications advise checking the assumptions about the data before selecting the bias adjustment method (Goh *et al*, 2017; Evans *et al*, 2018).

The issue of batch correction is further complicated by the fact that each technology faces different issues. Specifically, RNA‐seq batch effect adjustment requires approaches that address sequencing‐specific problems (Dillies *et al*, 2013). Similarly, MS methods in proteomics (e.g., data‐dependent acquisition—DDA, data‐independent acquisition—DIA, and tandem mass tag—TMT) also present several field‐specific challenges. First, there is the problem of peptide to protein inference (Clough *et al*, 2012; Choi *et al*, 2014; Rosenberger *et al*, 2014; Teo *et al*, 2015; Muntel *et al*, 2019). As protein quantities are inferred from the quantities of measured peptides or even fragment ions, one needs to decide at which level to correct the data. Second, it is known that missing values can be associated with technical factors (Karpievitch *et al*, 2012; Matafora *et al*, 2017). Finally, when dealing with experiments with large sample numbers, typically in the order of hundreds, one needs to account for MS signal drift.

Here, we discuss the application of established approaches for batch effect adjustment. We also look at the methods that address MS‐specific challenges. We start by providing an overview of the workflow and a definition of key terms for each step. In addition to considering batch effect assessment and adjustment, we summarize the best practices for assessing the improvements in data quality post‐correction. We also devote a section to the implications of missing values in relation to batch effects and potential pitfalls related to their imputation. We finish with a discussion and a future perspective of the presented approaches.

To facilitate the application to practical use cases, we illustrate all the relevant steps using three large‐scale DIA and two DDA studies. For these "case studies", we primarily rely on the largest of the five datasets (i.e., Aging mouse study; preprint: Williams *et al*, 2021) and refer to the others where appropriate. The data analyses we show are only for illustration purposes and are not intended for deriving new biological insights.

## Workflow overview

**Figure 1:**
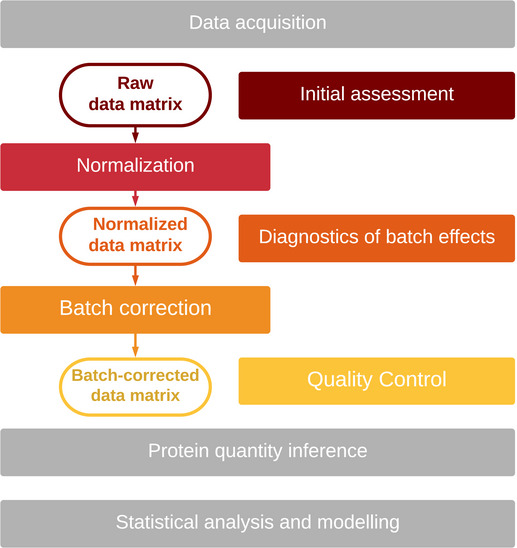
Batch effect processing workflow 1. Initial assessment evaluates whether batch effects are present in raw data. 2. Normalization brings all samples from the dataset to a common scale. 3. Diagnostics of batch effects in normalized data. This step determines whether further correction is required. 4. Batch effect correction addresses feature‐specific biases. 5. Quality control tests whether bias has been reduced while retaining meaningful signals.

The purpose of this article is to guide researchers working with large‐scale proteomic datasets toward minimizing bias and maximizing the robustness and reproducibility of results generated from such data. The workflow starts from a matrix of quantified features (e.g., transitions, peptides, or proteins) across multiple samples, here referred to as “raw data matrix” and finishes with "batch‐adjusted" data, which are ready for downstream analyses (e.g., differential expression or network inference). We split the workflow into five steps, shown in Fig 1, and describe each of the steps below.

In the context of this article, we will use the term “adjust for batch effects” when referring to the whole workflow and “correct for batch effects” when referring to the correction of normalized data (see Table 1).

We provide a checklist that summarizes the most important points of the protocol in Table 2. It is also important to stress that batch factors should be already considered in the experimental design phase, to ensure that the data are not biased beyond repair, something that can happen when biological groups are completely confounded with sample preparation batches (Hu *et al*, 2005; Gilad & Mizrahi‐Man, 2015). For an extensive discussion on experimental design, we refer the reader to previously published materials on the topic (Oberg & Vitek, 2009; Čuklina *et al*, 2020). Here, we assume that the experiment has been designed with appropriate randomization and blocking, ensuring the correctability of bias caused by batch effects.

**Table 2:** Batch effect processing checklist.

Step	Substeps
Experimental design^a^	Randomize samples in a balanced manner to prevent confounding of biological factors with batches (technical factors).
	Consider adding replicates if possible, for example: (a) add replication for each technical factor; (b) regularly inject a sample mix every few (e.g., 10–15, but the exact number will need to be adjusted depending on experimental conditions) samples for control; (c) incorporate a sample mix per batch.
	Record all technical factors, both plannable and occurring unexpectedly.
Initial assessment	Check whether the sample intensity distributions are consistent.
	Check the correlation of all sample pairs.
	If intensities or sample correlations differ, check whether the intensities show batch‐specific biases.
Normalization	Choose a normalization procedure, appropriate for biological background and data properties.
Diagnostics	Using diagnostic tools, determine whether batch effects persist in the data.
	Use quality control already at this step and skip the correction if it is not necessary.
	Tip: If the goal is to determine differentially expressed proteins, and the batch effects are discrete or linear, multi‐factor ANOVA on normalized data is a sound statistical approach. This will adjust for batch effects while simultaneously identifying differentially expressed proteins. Note, that "hits" or differentially expressed proteins identified with this approach are valid even if diagnostic tools indicate the presence of batch effects. For more details on ANOVA methods, refer to (Rice, 2006).
Batch effect correction	Choose batch effect correction procedure, appropriate for the biological background and data properties, especially those detected at the previous step.
	Repeat the diagnostic step.
	Assess the ultimate benefit with quality control.
Quality control	Compare correlation of samples within and between the batches. Pay special attention to replicate correlation, if these are available.
	Compare correlation of peptides within and between the proteins.

^a^ For details on experimental design, see (Čuklina *et al*, 2020).

In the accompanying “proBatch” package, we implemented several methods with proven utility in batch effect analysis and adjustment. We also provide tips for integrating other tools that might be useful in this context, and for making them compatible. proBatch is made available as a Bioconductor package (https://www.bioconductor.org/packages/release/bioc/html/proBatch.html) and a pre‐built Docker container (https://hub.docker.com/r/digitalproteomes/probatch), as well as a GitHub repository (https://github.com/symbioticMe/batch_effects_workflow_code) of the workflow with all code and data required to reproduce the case study analyses.

Extensive comparison of various methods has been published previously (Luo *et al*, 2010; Chawade *et al*, 2014), and here, we summarize the best practices from these papers, as well as reviews (Leek *et al*, 2010; Lazar *et al*, 2013) and application papers (Collins *et al*, 2017; Sajic *et al*, 2018), and turn them into principles that can guide the reader in choosing an appropriate methodology.

### Raw data matrix: choosing between protein/peptide/fragment level

This workflow starts with a raw data matrix, for which initial steps such as peptide‐spectrum matching, quantification, and FDR control have been completed. Data are assumed to be log‐transformed unless the variance stabilizing transformation (Durbin *et al*, 2002) is used. In the latter case, the data transformation is included in the normalization procedure.

We suggest performing batch effect adjustment on the peptide or fragment ion level, as this procedure alters feature abundances that are critical for protein quantity inference (Clough *et al*, 2012; Teo *et al*, 2015).

We also suggest that all detected peptides, including non‐proteotypic peptides and peptides with missed cleavages, should be kept into consideration during batch effect adjustment. Keeping all measurements is required to better evaluate the intensity distribution within each sample, which is critical for subsequent normalization and correction steps.

### Initial assessment

The goals of the initial assessment phase are to determine bias magnitude and sources and to select a normalization method. In most cases, the intensity distributions differ among samples. Comparing global quantitative properties such as sample medians or standard deviations helps with the choice of normalization methods and the identification of technical factors requiring further control.

Three approaches are particularly useful for initial assessment: (i) plotting the sample intensity average or median in order of MS measurement or technical batch, allows to estimate MS drift or discrete bias in each batch; (ii) boxplots allow to assess sample variance and outliers; and (iii) inter‐ vs. intrabatch sample correlation. A higher correlation of samples from the same batch compared with unrelated batches is a clear sign of bias. Optionally, a few proteins or peptides can be checked for signs of bias.

### Normalization

The goal of normalization is to bring all samples to the same scale to make them comparable. Commonly used methods of normalization are quantile normalization, median normalization, and z‐transformation. Two main considerations drive the choice of normalization method:

- Heterogeneity of the data: If samples are fairly similar, the bulk of the proteome does not change, and thus, techniques such as quantile normalization (Bolstad *et al*, 2003) can be used. In datasets in which the samples are substantially different (i.e., when a large fraction of the variables are either positively or negatively affected by the treatment) different methods, such as HMM‐assisted normalization can be used (Landfors *et al*, 2011). Additionally, if some samples are expected to have informative outliers (e.g., muscle tissue, in which a handful of proteins are several orders of magnitude more abundant than the rest of the proteome), methods that keep the relationship of outliers to the bulk proteome need to be used (Wang *et al*, 2021).
- Distribution of sample intensities: The initial assessment step, especially boxplots, indicates which level of correction is required: In most cases, shifting the means or medians is enough, but when variances differ substantially, these need to be brought to the same scale as well.

It should be noted that after normalization, no further data correction might be required. This can be determined with the diagnostic plots and quality control methods described below. If the results are satisfactory, keeping data manipulation minimal is advisable.

### Diagnostics of normalized data

While normalization makes the samples more comparable, it only aligns their global patterns. Therefore, batch effects affecting specific proteins or protein groups might still represent a major source of variance even after normalization. Thus, the diagnosis of batch effects is most informative when performed on normalized data.

The diagnostic approaches can be divided into proteome‐wide and peptide‐level approaches. The main approaches for proteome‐wide diagnostics are as follows:

- Hierarchical clustering is an algorithm that groups similar samples into a tree‐like structure called a dendrogram. Similar samples cluster together, and the driving cause of this similarity can be visualized by coloring the dendrogram by technical and biological factors. Hierarchical clustering is often combined with a heatmap, mapping quantitative values in the data matrix to colors which facilitates the assessment of patterns in the dataset.
- Principal Component Analysis (PCA) is a technique that identifies the leading directions of variation, known as principal components. The projection of data on two‐component axes visualizes sample proximity. Additional coloring of the samples by technical/biological factors, or by highlighting replicates, facilitates the interpretation of what drives sample proximity. This technique is particularly convenient to assess clustering by biological and technical factors or to check for replicate similarity. Visualization without sample point or label overlay effects works in our experience up to about 50–100 samples in a dataset.

One should be careful in interpreting proteome‐wide diagnostics because these methods were designed for data matrices without missing values. Proteomic datasets often contain missing values for technical or biological reasons. For more details, we refer the reader to Box 1.

In proteomics, peptide‐level diagnostics are as useful as proteome‐wide diagnostics. As in other high‐throughput measurements, individual features, in this case, peptides, are visualized to check for batch‐related bias. In proteomic datasets, spike‐in proteins or peptides can be added as controls. In most DIA datasets, iRT peptides (Escher *et al*, 2012), if added in precise concentrations, are well suited for individual feature diagnostics. It should be noted that individual peptides have a variety of different responses to various batch effects, so checking a handful of peptides is necessary, whether endogenous or spiked‐in.

Another reason to check individual peptides in proteomics is to examine the trends associated with sample running order. These trends might occur as MS signal deteriorates and require special correction approaches.

Note, that in proteomics, individual features are sometimes not peptides, but transitions or peptide groups. Thus, methods referred here as peptide‐level diagnostics are applicable to any feature‐level diagnostics.

### Batch effect correction

Diagnostics help to determine whether batch effect corrections are needed. While global sample patterns are corrected during normalization, batch effects affect specific features and feature groups, and that is the level on which they need to be corrected.

In proteomic datasets, two types of batch effects are frequently encountered, continuous and discrete. If batch effects are continuous, e.g., manifest as MS signal drift progressing from run to run during the sample measurement process, an order‐specific curve needs to be fitted, such as a LOESS fit, or by using any other continuous algorithm. Signal drifts are likely to occur in studies profiling hundreds of samples. This problem is more prominent in mass spectrometry as compared to next‐generation sequencing and is thus still relatively new to the research community.

Discrete batch effects manifest as feature‐specific shifts of each batch as a whole. Here, methods such as mean and median centering work very well. An advanced modification of the mean shift is provided by ComBat (Johnson *et al*, 2007) that uses a Bayesian framework which can be applied to proteomic data (Lee *et al*, 2019). However, ComBat requires that all features are represented in each of the batches. Therefore, especially in large‐scale proteomic datasets, applying ComBat might require the removal of a substantial number of peptides that happen to be missing in at least one batch, regardless of how small this batch is (see Box 1 for details). Thus, one should be very careful when choosing the method for batch effect correction.

### Quality control

The purpose of the quality control step is to determine whether the adjustment procedures—normalization and/or batch effect correction—have improved the data. At this step, the data after adjustment are compared with the raw data matrix. There are two types of criteria to evaluate the data quality: (i) removal of the bias (negative control) and (ii) improvement of the data (positive control).

Typically, bias is considered removed if the similarity between samples is no longer driven by technical factors. This means that neither hierarchical clustering nor PCA shows clustering by batch, and the correlation of samples from the same batch is no longer stronger than the correlation of unrelated samples. Also, individual features should not show batch‐related biases. Thus, comparison of diagnostic plots for raw and adjusted data serves as the negative control.

Proving improvement achieved by batch correction is much harder. It is common to take “improved clustering by biological condition” or “higher number of differentially expressed proteins” as a positive control and generally, as a sign of data quality enhancement. However, both criteria are subjective: It is impossible to know beforehand, whether biological groups are separable in the proteomic space, especially if only a subset of proteins changes while the bulk of the proteome does not. Similarly, it is not possible to predict whether higher sensitivity for differential expression comes at the expense of added false‐positive hits. Therefore, we do not recommend using these criteria to assess normalization or batch effect correction. As described above, the choice of the method should rather be based on the properties of the samples. In general, since batch adjustment removes a certain portion of variance, the coefficient of variation for peptides and proteins in replicated samples should decrease. This is especially true for spike‐in peptides or proteins that are added to samples in controlled quantities. A stronger positive control is the assessment of reproducibility, such as comparison of lists of differentially expressed proteins or regression/classification models derived from two or more sample sets belonging to different batches (Lazar *et al*, 2013). It is expected that in adjusted datasets, the resulting lists of differentially expressed proteins, or proteins providing optimal class separation, will be highly overlapping (Shabalin *et al*, 2008). If two sample sets are independently used for predictive modeling, the predictive performance of such models is also expected to be comparable in adjusted datasets (Luo *et al*, 2010). Note, however, that while this method generalizes well to studies with data acquired by different technologies (e.g., microarrays vs. RNA‐seq for transcriptomics or DIA vs. DDA for proteomics), it is restricted to fairly large datasets (several dozens, preferably, hundreds of samples), as predictions from small‐scale experiments tend to be unstable (underpowered).

Here, we also propose two positive control methods that do not rely on large sample size and are applicable to most proteomic experiments. The first is based on sample correlation. It is expected that the correlation between technical or biological replicate samples is higher than the correlation of unrelated samples. Particularly, the distribution of replicate correlations should be clearly shifted upwards, even though replicates might occasionally correlate less than some unrelated sample pairs, and this distinction should be strengthened by batch adjustment procedures. Following similar logic, other distance metrics can be used to assess sample proximity, which can also be visualized as improved clustering of replicated samples, seen on hierarchical clustering or PCA component plots. The latter method visualizes every sample in the experiment and thus is most suited to assess studies with up to 150 samples, while bigger sample sizes are harder to visualize. The second assessment method is specific for bottom‐up proteomics and makes use of peptide correlation. Correlation of unrelated peptides is expected to be close to zero, while peptides originating from the same protein are likely to be positively correlated. Since tens of thousands of peptides are routinely detected in modern high‐throughput proteomic experiments, improvements in this metric are a reliable readout of data quality following batch adjustment.

## Description of datasets used to illustrate the workflow

To illustrate the application of the workflow described above, we use five proteomic datasets (three acquired in DIA and two in DDA mode), described in Table 3.

**Table 3:** Dataset description.

Sample	Organism	Sample source	Sample‐to‐sample heterogeneity	Technical factors	Biological factors	Protein (peak groups/precursors) number	Number of samples	Dataset accession
InterLab study	Human	Cell culture	Very low: samples come from the same tissue cultures and differ only by few spike‐in peptides	Data acquisition sites Profiling days	None	4,077 (31,886)	229	PRIDE PXD004886
PanCancer study	Human	Blood	High: samples come from cancer patients and matched controls with different cancer localization	Protein digestion batch	Case / control Cancer localization	205 (1,360)	162	PRIDE PXD004998
Aging mouse study	Mouse	Liver tissue	Medium: samples come from population of inbred mice originating from two parental strains	Protein digestion batch MS batch MS drift	Strain Diet Age	3,940 (32,449)^a^	413	PRIDE PXD009160
TMT mouse study	Mouse	Liver	Medium: samples from a population of inbred mice originating from eight parental strains	Sample preparation batch MS batch MS drift	Strain Age	6,813(66,418)^a^	120	PRIDE PXD018886
Bariatric surgery study	Rat	Lymph	Medium: samples come from inbred rat population	Liquid handling robot	Gastric bypass vs. placebo surgery	302 (1,987)	68	MassIVE MSV000087519

^a^ Number of proteins and peptides before filtering for peptides with too many missing values.

The first study, called here "InterLab study", assessed the robustness of SWATH‐MS in a multi‐lab setting (Collins *et al*, 2017; Data ref: Collins *et al*, 2017). A set of 30 stable isotope labeled (SIL) peptides (Ebhardt *et al*, 2012), partitioned in five groups, was serially diluted in HEK293 cell lysate. The SIL peptides in the resulting samples spanned a concentration range from 12 amol to 10 pmol. These five sample sets were distributed to 11 laboratories worldwide for measurement by SWATH‐MS according to a predetermined schedule. Each of the samples was run on 3 separate days, with the exception of the 4th sample that was run three times on each day. In total, 229 samples were profiled. Thus, the technical covariates whose effect needed to be assessed were the data acquisition site and day. Note that due to the technical nature of the study, no biological signal needed to be identified. As only a small number of SIL peptides is different across these samples, all changes can be attributed to technical covariates, and therefore, the samples in this study can be treated as replicates. Within this manuscript, we analyze only the influence of the acquisition site as a batch factor.

The second study, named here "PanCancer study" (Sajic *et al*, 2018; Data ref: Sajic *et al*, 2018), profiled the blood plasma glycoproteome of a cohort of patients with five solid carcinomas and matched controls. In total, 155 blood plasma samples were collected. Protein digestion and glycopeptide enrichment were performed in 4 batches, several weeks apart. To account for sample preparation reproducibility, 7 biospecimens were replicated and allocated to a different batch. To control for intra‐sample variation caused by the sample preparation protocol, bovine fetuin‐B was spiked in equal amounts into each plasma sample. In total, 162 samples were measured (the validation cohort from the original manuscript is omitted from this analysis).

**Figure 2:**
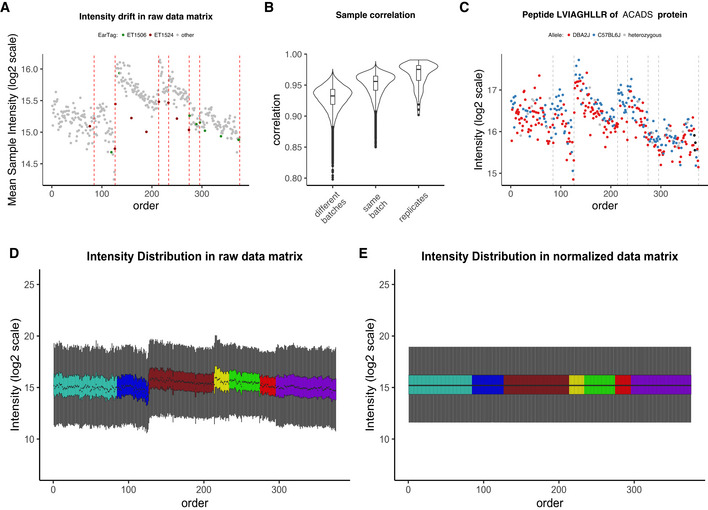
Initial assessment and normalization of the Aging mouse study (A) Mean intensity in raw peptide matrix vs. sample running order with repeatedly replicated samples shown in color. Vertical dotted lines indicate MS batch boundaries; (B) distribution of unadjusted sample intensity correlations—between batches, within batches, and in replicated samples; (C) bias in protein quantification: representative ACADS protein, the quantity of which follows the drift of the average sample intensity, preventing allele separation and QTL detection; (D) boxplots of sample intensities in raw, unnormalized peptide; (E) boxplots of sample intensities after quantile normalization. All plots represent peptide‐level data.

**Figure EV1:**
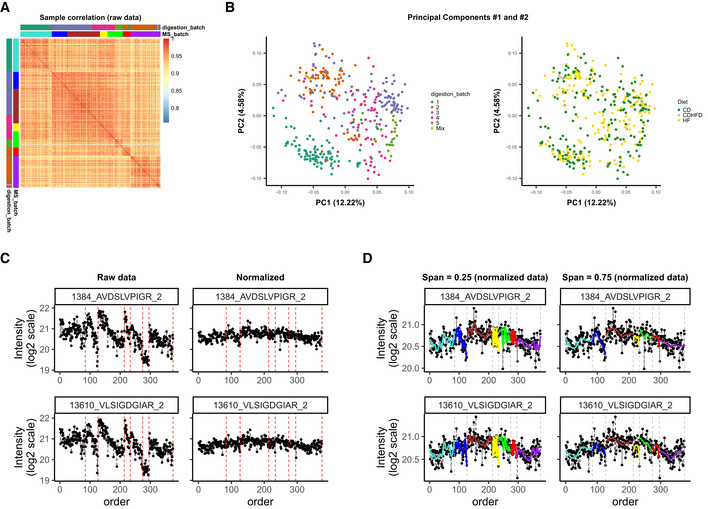
Batch effects in the Aging mouse study (A) Correlation of sample intensities indicates closer relationship between samples from the same batch; (B) Principal Components colored by digestion batch cluster together, but not the samples of mice on the same diet; (C) normalization removes a large fraction of variation, making samples more comparable, this is also seen at the level of individual peptides; (D) when fitting LOESS curve, span has to be chosen carefully: When too small, it will lead to overfitting and overcorrection.

The third dataset is called here the "Aging mouse study" (preprint: Williams *et al*, 2021; Data ref: Williams *et al*, 2021). In this study, 413 liver proteomes were measured from 341 individual mice from the BXD reference mouse population (Peirce *et al*, 2004) to identify changes associated with age. Similarly to prior BXD mice metabolic profiling experiments (Williams *et al*, 2016), genetically identical cohorts of animals were also subjected to either chow or high‐fat diet. The samples were randomized with respect to biological covariates (age, diet, sex), and samples from two mice with EarTags "ET1506" or "ET1524" were both injected 10 times at various intervals throughout the run to control for signal consistency. Additionally, a mix of samples was shot 3 times as control. In this experiment, two technical factors are known to affect the measurement. First, the samples were digested in five batches. Second, to compensate for signal deterioration, MS data acquisition was interrupted for machine cleaning and tuning, resulting in 7 mass spectrometry batches. These are shown as vertical lines in Fig 2. Except for replicates, samples were run in the same order of digestion batches (see Fig EV1A), so these two factors are mostly confounded (i.e., digestion and MS batch mostly overlap). Therefore, in our analysis we only correct for MS batch, unless noted otherwise. Given the particularly severe MS signal deterioration at the end of MS batch 2, the last 13 samples of this batch were profiled again as first samples of MS batch 3. In total, 375 proteome acquisitions were considered in this manuscript, while 38 acquisitions were discarded prior to analysis due to major acquisition failures. See also "Appendix" in supporting information for a summary of the original experimental setup of this study.

The fourth study, named here “TMT mouse study”, used data acquired from livers from 120 individuals from the Collaborative Cross reference mouse population, taken at 8 weeks of age (preprint: Keele *et al*, 2020; Data ref: Keele *et al*, 2020). Data were acquired in 12 TMT batches of 10 samples each, with a six‐month gap between batches 10 and 11. Batches 1–10 and 11–12 were prepared and run directly sequentially. The peptide measurement table from that paper was used as input for proBatch, and *cis*‐pepQTLs were calculated before and after proBatch.

The fifth study, named here “Bariatric surgery study” (Kaufman *et al*, 2019; Data ref: Kaufman *et al*, 2019), profiled N‐linked glycoproteomes from rat lymph before and after gastric bypass surgery. The cohort consisted of 68 lymph samples originating from rats before and after gastric bypass surgery (RYGB) or placebo surgery (SHAM). Samples were collected from rats before, 5, 10, and 21 days after the operation. The samples for this study were processed using a Versette automated liquid handling system (ThermoFisher Scientific) in a 96‐well plate format. Differences in the performances of Versette’s channels have introduced a “robot batch” into these data. The samples were measured in label‐free DDA mode, and glycopeptides were quantified using Progenesis (non‐linear dynamics).

All in all, these datasets are representative of various applications of large‐scale proteomic studies. They make use of different sample sources (i.e., cell cultures, patients, model organisms). They ask technical and biological questions about proteomes of varying complexity and present different degrees of sample‐to‐sample heterogeneity. In this respect, the InterLab study is very homogeneous, to the point that all the samples are essentially technical replicates. The PanCancer study is highly heterogeneous and comprises 205 proteins identified in samples originating from different hospitals and different tissues. Finally, the model‐organism‐based studies represent an intermediate case. On the one hand, its subjects were genetically related, but on the other hand, sampling introduces a certain amount of sample heterogeneity.

## Case studies

### Initial assessment

The main goal of the initial assessment is to set a baseline for the magnitude and nature of batch effects in a particular dataset. At this stage, the data matrix is “raw”, and the quantities are reported as measured, without any calibration, normalization, or correction with regard to their values in other samples.

Thus, it is essential to get a quick overview of the data by comparing global statistics, such as the average intensity, or correlation of samples and of few individual proteins with known expected abundance. In mass spectrometry, it is helpful to first plot these statistics by sample running order, as it is common for the measured signal to drift (e.g., due to deterioration of the LC and/or the MS). This is clearly seen in Fig 2A, where the average intensities of samples from the Aging mouse study are plotted vs. the sample running order. In this case, the intensity tended to deteriorate after 50–70 samples. The resulting interruptions for cleaning and calibrating the instrument determined the discrete mass spectrometry batches. As this type of bias is predictable, but cannot be entirely planned for in advance, it is particularly important to randomize the samples and to include replicates (for more details on sample replication in this dataset, see the “Description of datasets used to illustrate the workflow” section).

**Figure EV2:**
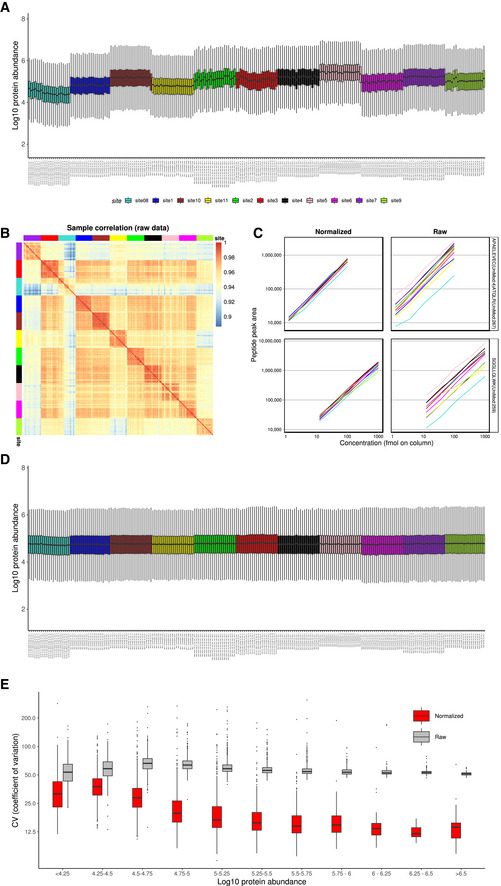
Batch effects in the InterLab study (A) Boxplots of raw protein intensity distribution in each sample colored by MS spectra acquisition site; (B) sample correlation heatmap for protein intensities, top row and left column colored by MS spectra acquisition sites, used as a quality control; (C) comparison of two spike‐in peptide quantification in raw and normalized data; (D) boxplots of median‐normalized protein intensity distribution in each sample colored by MS spectra acquisition site; (E) quality control by comparing coefficient of variation (CV) for proteins, binned by log10 abundance.

Not only do batch effects introduce shifts in the total sample intensity distributions, but they also lead to a spurious correlation between features (i.e., fragments, peptides, or proteins). It is common, for samples belonging to the same batch to have strong correlation (as seen in Figs 2B, EV1A, and EV2B). Often this correlation is not only stronger than the correlation of samples from different batches (“between batches”) but also stronger than the correlation of replicates, hence the importance of assessing correlation distributions as early as possible. Sample correlation can be visualized as a square heatmap (Aging mouse—Fig EV1A, InterLab study—Fig EV2B) or as a correlation distribution box/violinplot (Fig 2B). The former is preferred with smaller sample sizes (i.e., roughly < 150 samples) and the latter with large datasets, as it is not possible to assess replicate correlation in large datasets on the heatmap.

Optionally, one can complement the initial assessment with the analysis of a few specific features (peptides or proteins), for which prior information is known. Here, we plot a peptide from a representative protein *ACADS* with a known genetic sequence variant affecting its own expression at a quantitative trait locus (cis‐QTL), which can be seen as the bimodal expression separation on the plot according to the two possible ACADS alleles of the inbred strain. However, as seen in Fig 2C, the intensity drift, similar to that in Fig 2A, prevented the corresponding alleles in this BXD population, which descends from two strains, DBA/2J (red) and C75BL/6J (blue), from being separated based on expression level.

**Figure EV3:**
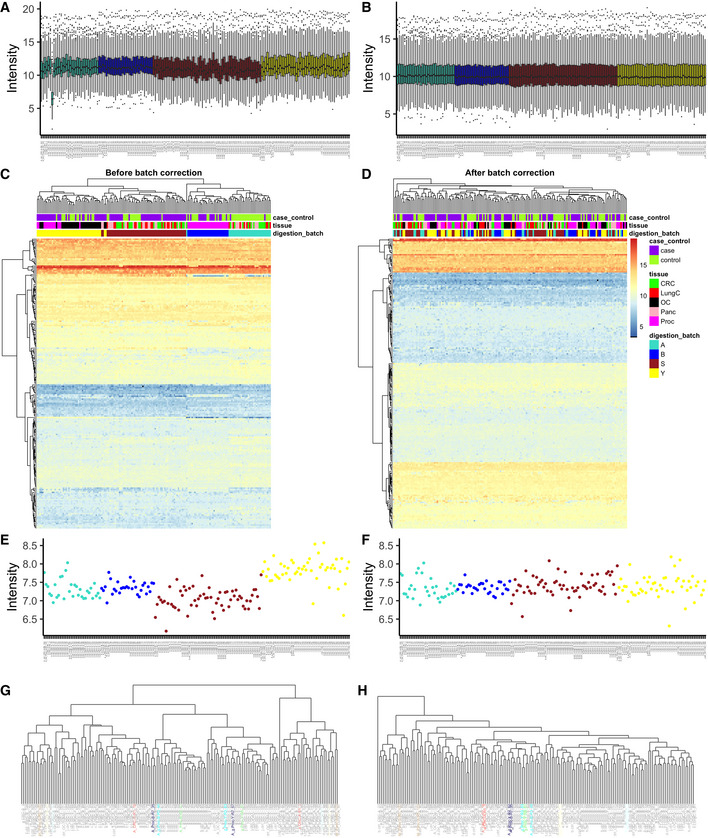
Batch effects in the PanCancer study (A) Boxplots of raw sample intensities colored by digestion batch; (B) boxplots of normalized sample intensities colored by digestion batch; (C) hierarchical clustering and heatmap of raw protein‐level data; (D) hierarchical clustering and heatmap of batch corrected protein‐level data; (E) intensity of spike‐in fetuin in raw data; (F) intensity of spike‐in fetuin in batch corrected data; (G) hierarchical clustering of normalized data with Manhattan distance, with replicated samples colored; (H) hierarchical clustering of batch corrected data with Manhattan distance, with replicated samples colored. All plots represent protein‐level data.

Last but not least, intensity boxplots are extremely powerful at this stage of the analysis, as they visualize in a single plot: median, quantiles, and outliers. This allows one to see whether there are batch‐specific intensity patterns such as shifts (as in the InterLab study, see Fig EV2A), or drifts (as in Fig 2D), or no evident batch‐associated patterns (PanCancer data; Fig EV3A). To detect the patterns, the samples should be sorted by running order or by batch (if the batches, such as digestion batches have been randomized prior to MS analysis). Note that in some cases, intensity patterns might be easier to spot on average intensity plot (compare to Fig 2A).

### Normalization

Normalization is an essential step in removing bias from the data as it brings the samples to the same scale, making the measured quantities comparable (Leek *et al*, 2010). As stated in the "Workflow overview" section, the choice of normalization method should take two factors into account: (i) heterogeneity, as assessed from previous knowledge, and (ii) global quantitative sample properties, e.g., mean/median/variance, as indicated by the initial assessment.

Quantile normalization (Bolstad *et al*, 2003) is applicable to a wide range of samples and was chosen for the aging mouse data, the PanCancer dataset, and the Bariatric surgery study. As shown in Figure 2D and E (and Fig EV3A and B), the intensity distributions after quantile normalization are very similar. This is desirable in experiments where the majority of features are not expected to change but can be problematic in experimental setups where outliers bear important information (Wang *et al*, 2021). The TMT mouse data were normalized outside of proBatch (preprint: Keele *et al*, 2020). Importantly, proBatch allows data to be taken up at different steps of the batch effects processing workflow and was used for this dataset only post‐normalization.

Median centering normalization only brings the medians to the same scale and thus is a “milder” approach. This normalization was chosen for the InterLab study and brought the peptide quantities measured at different sites much closer to each other (see Fig EV2C). In this dataset, shifting the medians to the same value was sufficient to remove a substantial portion of bias (see Fig EV2D, note that the correction on fragment level has residual median discrepancies on protein level). The resulting improvement in signal quality is also reflected in the improved protein quantification precision as illustrated by the reduction in coefficient of variation for the majority of proteins in the dataset (see Fig EV2E).

As shown in this example, normalization alone is sometimes the only required correction. In the median‐normalized InterLab study, the improvement of quantification of spike‐in peptides (Fig EV2C) and the decrease in protein coefficient of variation (Fig EV2E) serve as quality controls and indicate that further batch correction steps can be skipped.

This is of course not always the case, especially for larger datasets, and batch effect diagnostics should be used, as presented in the next section, to determine whether further correction steps are required.

### Diagnostics

**Figure 3:**
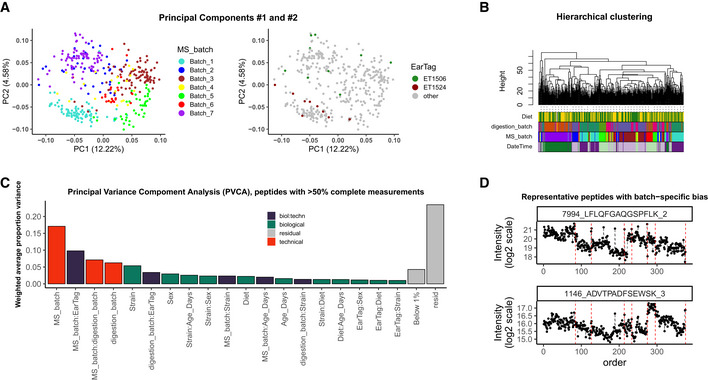
Diagnostics of batch effects: Aging mouse study (A) Principal Components #1 and #2 colored by MS batch (left) and replicates (right), percentage of variance in each PC shown in brackets. The effect of clustering by MS batch is dominating, but the replicated samples are closer to each other than just random samples; (B) hierarchical clustering of samples, with leaves colored by diet, digestion batch, MS batch, and date–time of sample acquisition is dominated by technical factors; (C) Principal Variance Component Analysis of peptides, detected in >50% of samples demonstrates, that the technical factors, such as MS batch, digestion batch, and their combination, have a profound effect on the data, while biological factors such as strain, sex, and age account for a much smaller fraction of variance; (D) peptide‐level plots for two iRT peptides demonstrate that batch effect manifests also as MS signal drift that requires correction.

**Figure 4:**
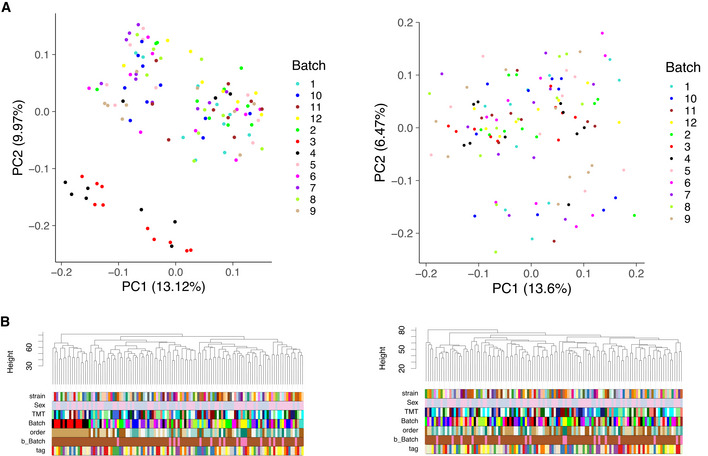
Batch effects in the TMT mouse study (A) PCA and (B) Hierarchical clustering in matrix of peptides with complete measurements before (left) and after (right) correction.

Normalization harmonizes overall sample intensities; however, batch effects can still be present at the level of specific features and bias the quantities of many peptides and proteins. Various methods can be used to assess the extent of remaining bias in the data. Most methods characterize the dataset as a whole, thus working on the proteome level. Methods such as Principal Components Analysis (PCA) and Hierarchical Clustering are the best established. For instance, in the Aging mouse study, the following patterns became apparent (see Fig 3A): First, we see a strong clustering of samples by mass spectrometry batch; second, while replicates tend to be close, they are not necessarily the samples with the smallest distance to each other. An overlaid visualization of other factors, such as digestion batch, diet, or acquisition date, can be found in Fig EV1B. Also for the TMT mouse study, PCA shows strong clustering by batch (TMT batch, see Fig 4A).

Hierarchical clustering, in contrast, allows one to visualize multiple factors at once (Fig 3B). Overall, the same patterns identified in the PCA plots are confirmed as follows: Clustering is driven primarily by MS and digestion batch, while the effect of diet, a dominant biological factor, is not as strong. Similarly, in the PanCancer study (Fig EV3C and D, top parts), we see that the digestion batch is the key clustering factor, and also in TMT mouse study, TMT batch (Batch) drives the clustering of samples (Fig 4B).

**Figure EV4:**
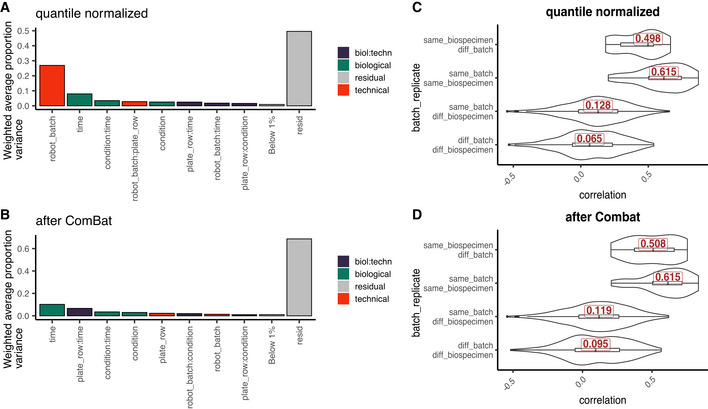
Batch effects in the Bariatric surgery study (A) Principal Variance Component Analysis shows a clear effect of the liquid handling robot’s channels (robot_batch (plate rows A and B vs. C–F)). (B) This technical factor is efficiently removed after ComBat batch correction. (C) Distribution of correlations between and within batches for different time points from the same animal and different animals prior to and (D) post‐batch correction. Peptide intensities in C and D are normalized to pre‐surgery intensity values. The procedure improves the correlation of samples coming from the same animal at different time points which can be used as pseudo‐biological replicates in this study.

Principal Variance Components Analysis (PVCA) transforms the intuition of visualization brought about by PCA and Hierarchical Clustering into numbers. The weights of each factor in each Principal Component are combined, thus providing a concise summary of variance distribution between biological and technical factors and their combination. In the aging mouse dataset, we see in Fig 3C that technical factors such as MS batch, digestion batch, and their combination are leading drivers of variance. Whereas biological factors, such as diet, sex, and age, are much less prominent. It should also be noted that a substantial fraction of variance is “residual” and cannot be explained by the annotated sample characteristics. PVCA also shows a clear batch effect from the liquid handling robot for the Bariatric surgery study (see Fig EV4A).

It is important to point out that PCA, hierarchical clustering, and PVCA require matrices with complete feature measurements, or with imputed missing values. Since in MS proteomic missing values are often batch‐specific, this might lead to an overestimate of the batch‐related clustering (variance explained) and to over‐pessimistic assessment of data quality (see also Box 1).

In addition to proteome‐wide diagnostics, it is often informative to visualize a few individual features to assess for the presence of feature‐level bias. Spike‐in peptides and proteins are particularly handy for this type of diagnostics. We illustrate this in the aging mouse data using iRT peptides that were spiked at constant quantities in all samples. In Fig 3D, one can see, that despite the normalization, batch‐specific bias still affects the signal. Moreover, this bias is order‐related and manifests differently for each of the peptides (e.g., in samples 127–212 of MS_batch 3). In the PanCancer study, we see that protein quantities are biased by batch effect on the example of the spiked‐in bovine protein fetuin‐B (Fig EV3E and F). In contrast to the Aging mouse study, here there is no order‐related drift, but rather each batch mean is shifted. In conclusion, feature‐level bias can be very different in each dataset and checking several representative peptides can help understand the exact nature of the bias.

Together, proteome‐level and feature‐level diagnostics guide the choice of appropriate batch correction procedure.

### Batch effect correction

The goal of batch effect correction is to alleviate the residual bias after normalization. In most cases, normalization significantly reduces the unwanted variance (see representative peptides in Fig EV1C) and, as in the InterLab study, might sometimes be the only required adjustment. However, in many cases, the batch effect diagnostic procedures described in the previous section will reveal that individual features are differentially affected by batch effects even after normalization (Figs 3D and EV3E). Batch effect correction is typically applied at the feature level.

Feature‐level bias in proteomics can have different roots. In the aging mouse data, the peptides manifest an order‐specific, continuous bias, while data in the PanCancer exhibit discrete shifts. The latter can be corrected with methods established by the genomic community, whereas the former is typical of large MS‐based proteomic experiments and will therefore be discussed in more detail in the following paragraphs.

In MS‐based proteomics, samples are analyzed sequentially, typically using an online chromatographic system directly coupled to a mass spectrometer. Various components of this system are susceptible to degradation of performances over time (e.g., changes in the properties of the chromatographic material, emitter degradation, contamination of MS lenses, mass calibration drift). Various factors, such as sample quality and composition, can contribute to the speed of this degradation. This is particularly relevant in large‐scale studies.

**Figure 5:**
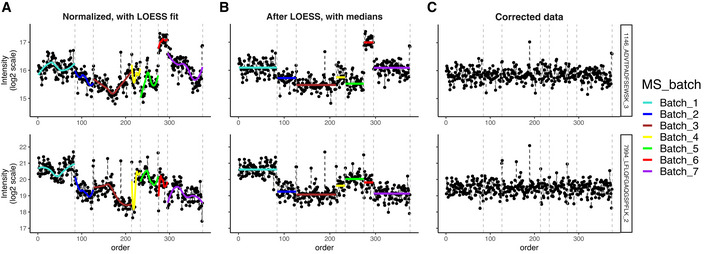
Two‐step correction of batch effects (A) Fitting a LOESS curve for every peptide in each batch and subtracting the fit; (B) using medians for correction of the residual discrete batch effect; (C) the corrected data are more uniform and can now be used for downstream analysis. Data from the Aging mouse study.

To address this complex bias, we have developed a two‐step batch correction procedure shown for the aging mouse in Fig 5. In the first step, MS signal drift is corrected based on non‐linear curve fitting. In the second step, remaining discrete batch effects are addressed using a discrete batch correction procedure (e.g., median centering, ComBat).

As a curve‐fitting algorithm for the first step, we have chosen LOESS, as it combines computational simplicity with relative flexibility of the fit characteristics. The procedure runs as follows: For each peptide and each batch, a unique curve is fit (Fig 5A) and then subtracted from the normalized intensity value in each sample, leading to measurements, whose median is different in each batch (Fig 5B). Note, that LOESS has a parameter "span": Smaller span fits minute details, while when the span is too big, some of the trends get missed, meaning each time the span has to be adjusted to each dataset individually (see Fig EV1D). Alternatively, other algorithms, such as SVM and Random Forest, can be used to correct for the MS signal drift (Shen *et al*, 2016; preprint: Luan *et al*, 2018). For the second step, the residual discrete batch effects were removed by median centering (Fig 5C).

Datasets that do not exhibit MS signal drift require only discrete batch correction—second step of the aging mouse dataset. Here, the PanCancer (Fig EV3C–F) and TMT mouse (Fig 4A and B) were corrected in a single step using median centering. Similarly, in the Bariatric surgery dataset (Fig EV4A and B) the liquid handling robot represented the main source of discrete bias and was corrected in a single step using ComBat (Johnson *et al*, 2007).

When several batch factors affect the data (as diagnosed by PCA, hierarchical clustering, and/or PVCA), all factors should be accounted for during correction. This typically means that batch factors get combined. This, however, is not always possible (e.g., batches become too small). In the Aging mouse study, for instance, the two main technical factors, MS batch and digestion batch, were highly confounded. Thus, we opted to only correct for the MS batch.

In conclusion, batch effect correction removes variance from known, annotated batch factors. Whether the adjustment procedures—normalization and batch effect correction—improved the data signal, remains, however, to be determined by a quality control step.

### Quality control

The main goal of the quality control step is to determine whether the data quality has improved.

Examples of negative controls are the plots discussed in the diagnostic sections such as individual peptide plots, PCA, hierarchical clustering, or PVCA. In most cases, these show that samples are no longer affected by batch‐specific patterns (e.g., PanCancer study hierarchical clustering in Fig EV3C and D and spike‐in protein in Fig EV3E and F).

**Figure 6:**
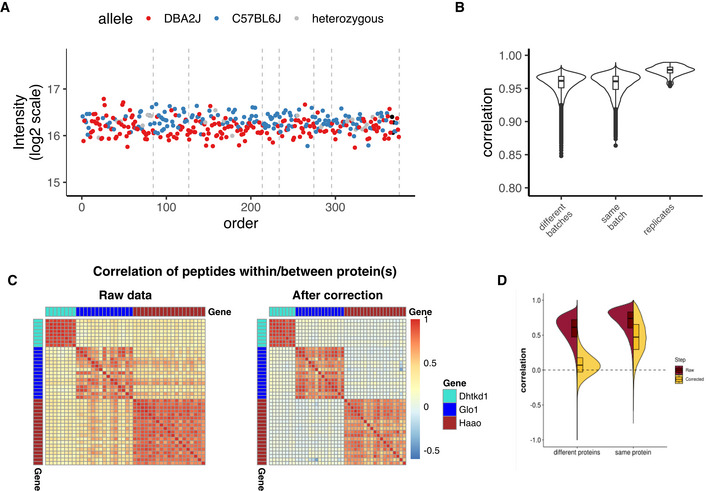
Quality control of batch effect correction (A) Representative "Acads" protein QTL that in corrected data demonstrates a clear improvement in allele separation; (B) distribution of sample correlations between batches, within batches, and in replicated samples for corrected data, compare to Fig 2B; (C) heatmaps of peptide correlation for the proteins DHTKD1, GLO1, and HAAO, before and after correction: Correlation is positive for all peptides in the raw matrix, while after batch correction, the correlation of unrelated peptides becomes close to zero; (D) distribution of peptide correlation in raw data (brown) and in batch corrected data (yellow) for peptides from different proteins and peptides from the same protein: While same‐protein peptide correlation is always higher than the correlation of unrelated peptides, the correlation of unrelated peptides approaches zero only after the correction.

Depending on the experimental setup, one can sometimes judge the data improvement through the presence of a better biological signal. In the Aging mouse study, this can be achieved via mapping protein quantitative trait loci (pQTL), particularly *cis*‐QTLs (i.e., alleles of specific genes have DNA variants which affect their own protein’s expression level, which is tested on peptide level). As seen in Fig 6A, certain proteins become clearly easier to separate according to their variant allele and less biased than before the bias adjustment (compare to Fig 2C). As a result, there is a clear increase in pQTL detection sensitivity: 255 *cis*‐pQTLs passed the significance threshold in the raw data matrix, and extra 133 *cis*‐pQTLs have been detected after batch effect adjustment—100 after normalization and 33 additional *cis*‐pQTLs after batch effect correction. On peptide level, this corresponds to 993 peptide‐level pQTLs in raw data, additional 405 peptides after normalization and 352 after batch effect correction. Similarly, for the TMT mouse dataset 3,306 cis‐pepQTLs were detected at LOD ≥ 4 among the 8,774 peptides with complete measurements in the normalized data, with an additional 109 cis‐pepQTLs detected after correction for TMT batch factor. Note, that for the aging mouse dataset, normalization seems to alleviate most of the bias, as was already demonstrated in Fig EV1C. This effect is quite common and is the reason why the batch effect correction step was not performed in the InterLab study. Skipping batch effect correction is also reasonable, when the key question is “which proteins are differentially expressed”, which is best address by ANOVA, or when it is not clear which technical factor should be corrected for (e.g., diagnostic plots do not show a clear clustering pattern and the weights of technical factors on PVCA are low). However, when biological groups are not confounded with technical factors, batch correction reduces noise and should improve the signal quality.

On the other hand, the sample and feature proximity methods we proposed in the “Quality control” section can be applied in almost any proteomic experimental context and thus are a more universal approach to quality control.

Sample proximity measured as correlation distributions for intrabatch vs. unrelated samples, and for replicates vs. all other samples can be seen for the aging mouse dataset pre‐ and post‐adjustment in Figs 2B and 6B and for the Bariatric surgery study in Fig EV4C and D. This approach and considerations can also be applied with different distance measures and visualizations. Fig EV3G and H demonstrate this concept on the PanCancer data using Manhattan distance and a hierarchical clustering representation. These proximity‐based quality controls are universally applicable to any dataset with replicated samples and batches.

The second method is based on assessing the proximity of feature sets. In bottom‐up proteomics, these are peptides or fragment ions. Similarly to the previous method, the distance between related peptides (i.e., belonging to a common protein) is expected to be smaller than the distance between unrelated ones. This effect is true not only for selected proteins (Fig 6C) but also holds true for the whole proteome (Fig 6D). The correlation of unrelated peptides, however, gets much closer to zero after the correction. Hence, before batch adjustment most of the peptide correlation was spurious.

In summary, through a combination of positive and negative quality controls, it is possible to assess improvements in data quality. It should, however, be stressed that quality control methods are not criteria for choosing batch adjustment methods. Their purpose is rather to provide metrics to control the quality of the data before and after adjustment.

## Computational tools and analysis workflow for future applications

To facilitate the practical application of the steps and principles described here, we have developed an R package, named "proBatch" and made it available in Bioconductor (https://www.bioconductor.org/packages/release/bioc/html/proBatch.html). The "proBatch" package wraps multiple established techniques for data transformation and visualization with proven utility in the control of batch effects.

The package has been designed based on the following principles to facilitate the construction of batch effect adjustment pipelines:

For each step of the workflow, one or more functions are provided.

The input to each function is standardized to a feature measurement table (e.g., peptide, protein, fragment, transition) and a sample annotation table with technical and biological factors.

Consistent visual representation of factors at all steps of the analysis (e.g., color scheme defined once per analysis).

Note that we have also deliberately avoided the inclusion of a "single‐click" batch adjustment function that would combine normalization and correction. As explained above, the choice of the specific algorithm for normalization and batch correction depends on the properties of a particular dataset, and thus, keeping these functions as separate exchangeable modules should prompt the researcher to check the properties of the data and the goals of the analysis and ultimately make an informed choice of appropriate batch adjustment methods.

To demonstrate how the package can be applied to different experimental setups, and how different parameters of the study can be adjusted, we make the code used for the analyses of the five case studies presented in this manuscript available as a GitHub repository (https://github.com/symbioticMe/batch_effects_workflow_code) of the workflow. An accompanying Docker container containing all the tools required to replicate the analyses is made available on DockerHub as a proBatch Docker container (https://hub.docker.com/r/digitalproteomes/probatch).

## Conclusions

The meaningful analysis of data generated by large‐scale proteomic studies, made possible by recent technological advances in data collection, is critically dependent on the statistical power required for systems biology and translational medicine studies. However, great power comes with batch effect baggage and requires specialized tools to handle this problem.

It is sometimes argued that batch effects should not be corrected, but rather incorporated into the downstream analysis. We agree that accounting for batch effects in differential expression analysis using ANOVA is backed up with decades of statistical research and practice. However, ANOVA has several important disadvantages. First, ANOVA is best suited to adjust for batch effects on fragment or peptide level, as this is the level where batch effects manifest, while in practice researchers are interested in protein‐level protein expression differences; second, ANOVA adjusts for linear or discrete batch effects, while MS‐based proteomics is often affected by non‐linear signal drift; and finally, when using protein quantities outside of differential expression analyses, such as in protein–protein correlation comparison in molecular networks, adjusting for batch effects with classical statistic approaches can be difficult. This explains why most large‐scale studies opt for adjusted batch effects and proceed with a “batch‐free” dataset.

Another common advice is to not correct batch effects to avoid “flattening” the biological signal. This can occur when the biological groups are confounded with the batch factors. However, confounding is a serious experimental design error and can be prevented by balancing the biological groups between the expected batches. On the other hand, to account for ill‐defined batches (e.g., MS batches) samples can be randomized. "Overcorrection'' can also potentially occur with small‐sized batches, where fitting a non‐linear curve or accurately estimating the mean and the variance becomes difficult. Hence, it has been suggested that batches should contain at least 25 samples (Alter *et al*, 2000; Benito *et al*, 2004). To check whether small batches add too much noise, one can test the stability of the significant hits with and without the batch in question. In most cases, however, the strength of the signal is sufficiently assessed at the quality control step, described in detail in this manuscript.

While most batch effect methods are similar across "omics" fields, missing values are more typical in MS proteomics than transcriptomics or genomics data. Missing values are often batch‐associated (Karpievitch *et al*, 2012) and can cause established clustering and correction methods to fail. For example, missing values often lead to “uncorrectable” batch effects seen as batch clusters in PCA. This, however, is often due to filling the batch‐specific missing values with zeros or small random numbers. Other methods, such as hierarchical clustering or ComBat (Johnson *et al*, 2007), could potentially be adapted to account for the sparsity of proteomic data. Even so, proteomics could benefit from the development of more methods robust against missing data.

Specialized proteomic applications could also benefit from further batch adjustment methodological developments. Here, we presented a workflow and applied it to DIA and DDA (label free and TMT based) data. The workflow relies on having a quantitative data matrix with few assumptions on its structure and is applicable to most quantitative proteomic workflows. However, proteomic applications incorporating affinity purification, size exclusion chromatography, PTM‐centric protocols, or subcellular fractionation are typically more heterogeneous. These dataset types will likely require additional efforts for batch effect characterization and correction.

While searching for publicly available datasets to present as case studies in this manuscript, we also noted that many of the available studies have been deposited with little to none of the technical meta‐data required to perform batch correction. We believe that this limits the re‐usability of proteomic data and would like to encourage future studies to include this information.

In conclusion, mass spectrometry‐based proteomics has come a long way and is continuing to evolve. As throughput and reproducibility increase, so do batch effect‐related issues. Hence, we expect experimental design and batch effect correction methods to also grow in importance and to take center stage in large‐scale proteomic applications such as clinical proteomics and systems biology.

## Author contributions

JČ and PGAP designed the analysis workflow and wrote the manuscript; EGW introduced multiple components in the workflow and made major contributions to the manuscript outline and text. JČ, CHL, EGW, VSS, and GRK performed data analysis. JČ, CHL, and PGAP implemented back‐end R functionality; EGW helped with the code testing. EGW, TS, BCC, and GRK provided proteomic data and contributed to workflow conceptualization. FW, SG, and BW provided proteomic data measurements and analysis. MRM provided critical input on the project and assisted in analysis pipeline design. MRM and RA commented and edited the manuscript. PGAP and RA supervised the study.

## Conflict of interest

The authors declare that they have no conflict of interest.

Box 1 **Missing values**

**Figure EV5:**
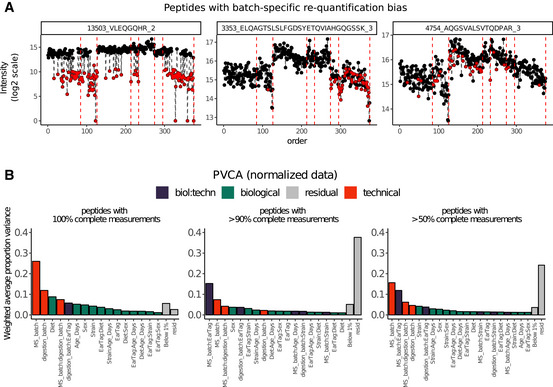
Missing values and batch effects in the Aging mouse study (A) Re‐quantification of elution traces can pick up batch‐specific noise that can be drastically different (left panel) or indistinguishable (middle and right panel) from confidently identified peptide fragments, meaning that values inferred should be treated with extreme caution. Black points are regular quantifications, red points are re‐quantifications; (B) PVCA can only be applied to complete matrices, and thus, missing values need to be inferred. This makes this method highly sensitive to missing values inference (here filled with 0). Depending on the completeness cutoff, variance distribution across technical and biological factors varies substantially. Note that when peptides with missing values are used in the analysis (panels center and right), a substantial portion of variance is attributed to "resid" (residual), meaning that this variance cannot be associated with any of known factors, indicating that missing value distribution is at least partially random.

**Figure EV6:**
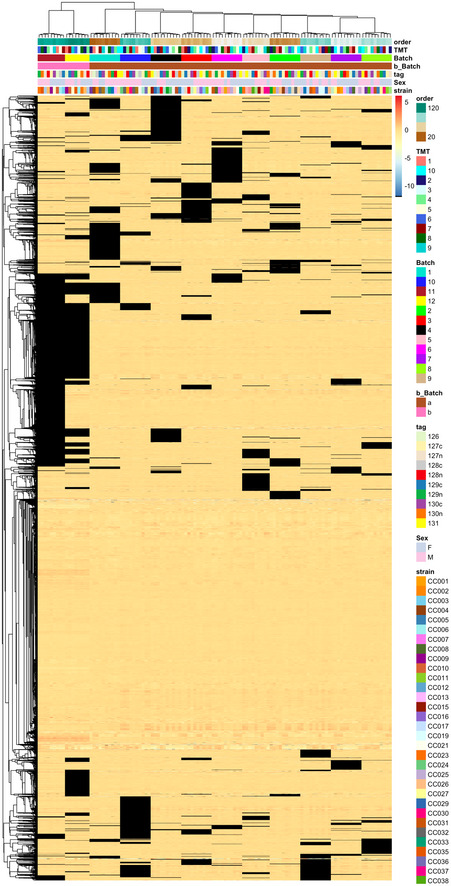
Missing values in the TMT mouse study Heatmap of peptides with up to 30% missing values, filled with minimal values (black) shows that the missing values are primarily associated with TMT batch and this drives the clustering; compare to complete data matrix in Fig 4 in which batches 3 and 4 cluster together.

Proteomic experiments now routinely profile hundreds or thousands of proteins across hundreds of samples. However, detecting all proteins without missing values across the whole dataset is not yet feasible. The patterns of "missingness" are known to be batch‐specific (Karpievitch *et al*, 2012), and some workflows are susceptible to a rapid inflation of missing values as the number of batches increases (Brenes *et al*, 2019). This is also true for the largest datasets of this manuscript: aging mouse DIA and TMT datasets (see Box 1 Figure, Figs EV5 and EV6 for details).

It should be noted, that even though "missingness" for low‐abundant peptides is more common (i.e., an issue related to the dynamic range and sensitivity of the mass spectrometer), this problem can also arise due to fundamental peptide interference regardless of their abundance or the acquisition parameters.

Missing values can also affect batch effect correction methodologies. For instance, the current implementation of ComBat (Johnson *et al*, 2007) does not work if a peptide is missing in one batch. One possible solution is to remove all peptides with missing values before the batch correction (Lee *et al*, 2019). However, this may lead to loss of valuable quantitative information. Thus, methods which are more robust to missing data, such as median centering, can sometimes be better suited for proteomic data.

Missing values are often imputed, by filling them with zeros, random small values (Tyanova *et al*, 2016) or re‐quantification of elution traces (Röst *et al*, 2016). Such imputation, however, can introduce bias that is batch‐ or peptide‐specific, as seen in Figs EV5A and EV6. In turn, this skews batch effect diagnostic methods, such as hierarchical clustering, PCA, or PVCA. In these cases, batch effect assessment will be biased, as the clustering pattern will be driven by missing values (Fig EV5A). One can estimate this effect by varying the fraction of missing values and assessing to what extent the batch effects are driven by consistently quantified peptides vs. missing values containing ones (Fig EV5B).

More importantly, imputed values bias the analysis past the batch effect adjustment stage. As shown in Box 1 Figure B and C, if re‐quantifications ("with requants") values inferred from MS elution traces are used, the correlation within batches seems higher than the correlation of replicates, while this problem is not observed when imputation is not used ("no requants"). Protein inference is also affected by the imputation on lower levels.

Finally, provided that there are enough confidently quantified values, many downstream analysis techniques, such as differential expression or protein correlation analyses, can handle missing values. We therefore advise to avoid imputation, or at least suggest to perform it after batch correction whenever possible.

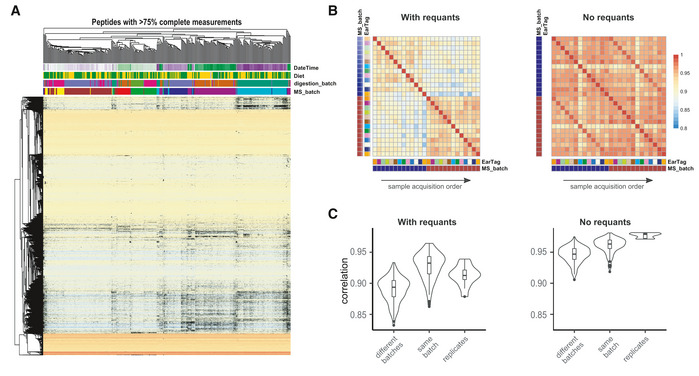

Box Figure 1. The problem of missing values in batch effect diagnosis and correction: Aging mouse study. (A) Hierarchical clustering and heatmap of normalized data; missing values shown in black. The missing values are non‐randomly associated with the batch; (B) heatmap of selected sample correlation: Stronger correlation of samples within Batch 2 (blue) and Batch 3 (brown) is visible in the data with "requants", and replicate correlation is much more prominent in the data without "requants"; (C) distribution of selected sample correlation: same effect, as in (B) showing the distribution of sample correlation.

## Supporting information



Appendix

Expanded View Figures PDF
