# Supplementary material for: Diagnostics and correction of batch effects in large‐scale proteomic studies: a tutorial
Source: Mol Syst Biol. 2021 Aug 25;17(8):e10240. doi: 10.15252/msb.202110240 (PMC8447595; doi:10.15252/msb.202110240)
Supplement: Supplementary file 1 — Appendix [file MSB-17-e10240-s002.docx]

# Table of contents

[Summary of the original experimental setup described in the Aging Mouse study (Williams *et al*, 2021) 1](#_Toc77252134)

[Mouse handling 1](#_Toc77252135)

[QTL detection methodology 2](#_Toc77252136)

#

## Summary of the original experimental setup described in the Aging Mouse study (Williams *et al*, 2021)

To facilitate better understanding the potential sources of biases and of the QTL analysis procedure, we provide a short summary of the relevant sections from the methods used in the original study.

### Mouse handling

The 341 mice (21 male, 320 female) used for the proteomics data were from 57 different, but related, inbred wildtype strains of the BXD family were raised at the University of Tennessee Health Science Center (UTHSC) with SPF conditions approved by the University of Tennessee Health Science Center’s Animal Care and Use Committee. Technical and biological replicates are described in the "Datasets description" section. Blinding and cohort randomization are not feasible for this type of study, as the genotypes must be known to perform QTL mapping. Animals were checked daily for signs of moribundity and if necessary euthanized according to the point system criteria determined by the NIH’s Guidelines for the Care and Use of Laboratory Animals. Liver tissue was exsanguinated, frozen in liquid nitrogen, and pulverized before being processed for proteomics. Full details on the animal care and sample processing are published with the dataset.

### QTL detection methodology

QTLs were calculated using the R package "qtl", version 1.42-8 using strain-averaged data from all BXD individuals. The same exact individuals were used for all batch effects analyses. A protein’s QTL was determined to be a "cis acting" QTL if its peak location was within 10 megabases of the known position of its gene using the position from mouse genome assembly version mm10. QTL LOD scores were calculated using the 2019 build of the BXD genotypes using the Haley-Knott method and non-parametric (np) model (i.e. scanone(inputdata, pheno.col=[each_protein_index], method="hk", model="np")).
