# Supplementary material for: Diagnostics and correction of batch effects in large‐scale proteomic studies: a tutorial
Source: Mol Syst Biol. 2021 Aug 25;17(8):e10240. doi: 10.15252/msb.202110240 (PMC8447595; doi:10.15252/msb.202110240)
Supplement: Supplementary file 2 — Expanded View Figures PDF [file MSB-17-e10240-s001.pdf]

Expanded View Figures

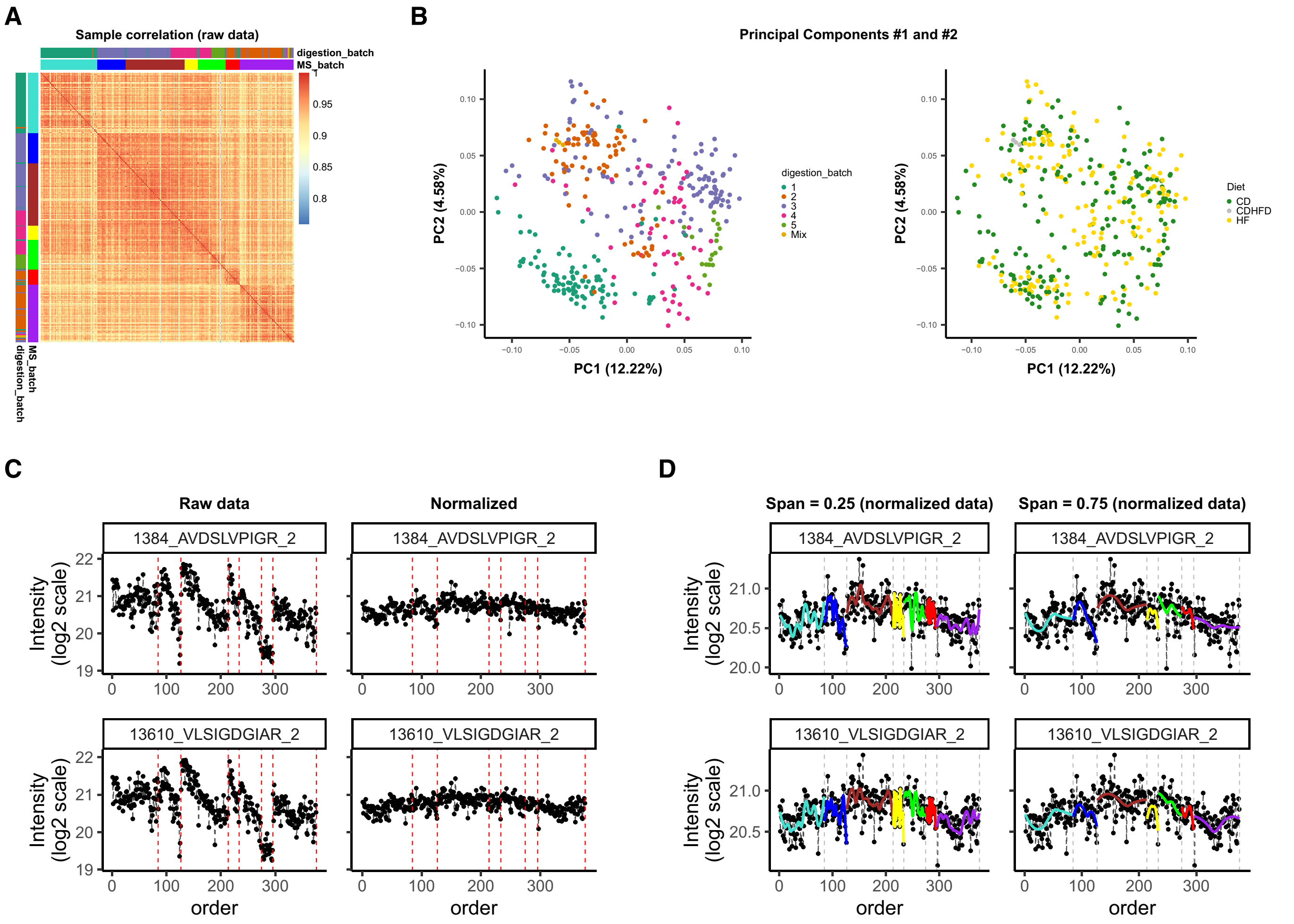

Figure EV1. Batch effects in the Aging mouse study.

(A) Correlation of sample intensities indicates closer relationship between samples from the same batch; (B) Principal Components colored by digestion batch cluster together, but not the samples of mice on the same diet; (C) normalization removes a large fraction of variation, making samples more comparable, this is also seen at the level of individual peptides; (D) when fitting LOESS curve, span has to be chosen carefully: When too small, it will lead to overfitting and overcorrection.

Figure EV2. Batch effects in the InterLab study.

(A) Boxplots of raw protein intensity distribution in each sample colored by MS spectra acquisition site; (B) sample correlation heatmap for protein intensities, top row and left column colored by MS spectra acquisition sites, used as a quality control; (C) comparison of two spike-in peptide quantification in raw and normalized data; (D) boxplots of median-normalized protein intensity distribution in each sample colored by MS spectra acquisition site; (E) quality control by comparing coefficient of variation (CV) for proteins, binned by log10 abundance.

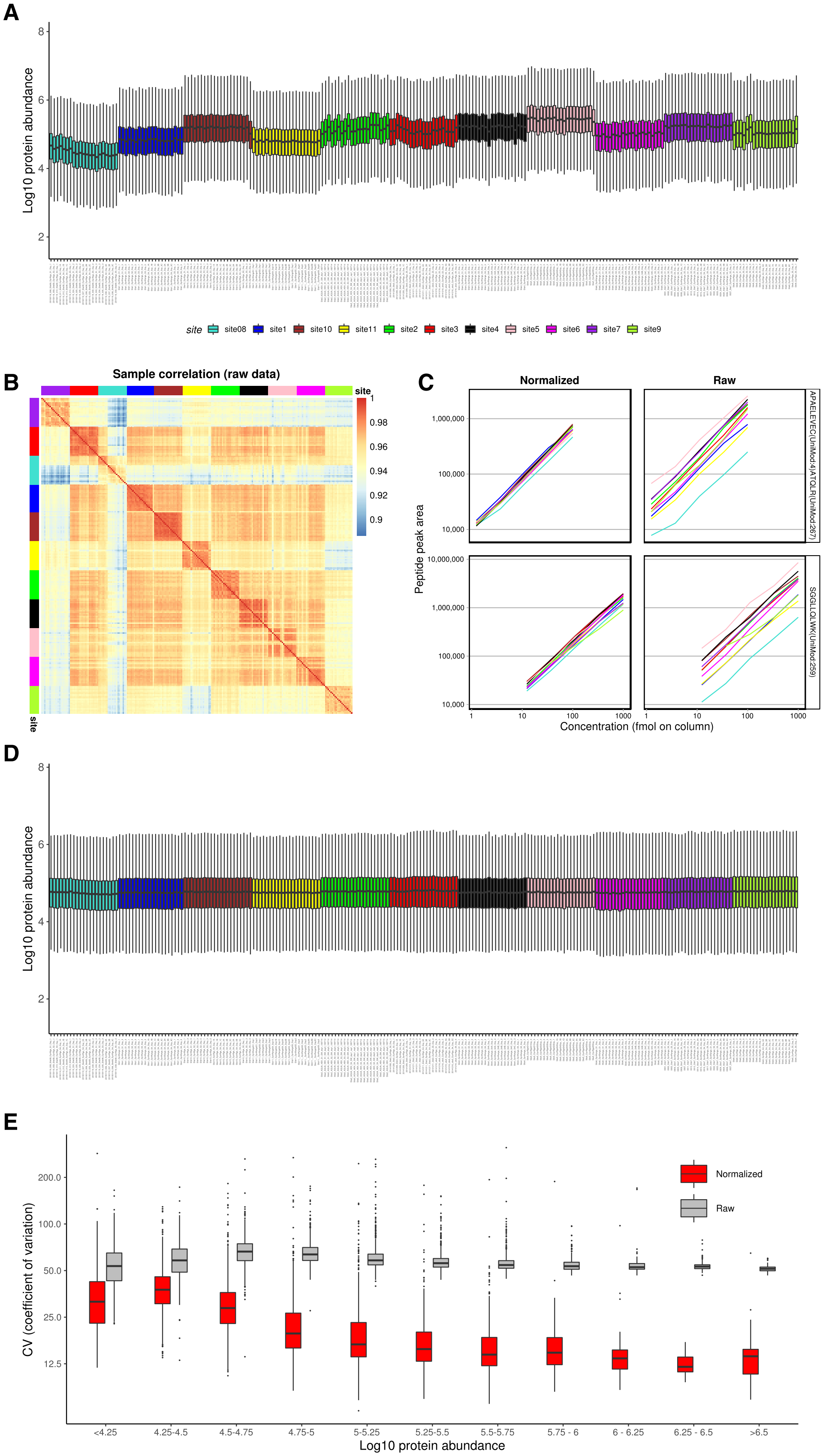

Figure EV2.

 © 2021 The Authors

Figure EV3. Batch effects in the PanCancer study.

(A) Boxplots of raw sample intensities colored by digestion batch; (B) boxplots of normalized sample intensities colored by digestion batch; (C) hierarchical clustering and heatmap of raw protein-level data; (D) hierarchical clustering and heatmap of batch corrected protein-level data; (E) intensity of spike-in fetuin in raw data; (F) intensity of spike-in fetuin in batch corrected data; (G) hierarchical clustering of normalized data with Manhattan distance, with replicated samples colored; (H) hierarchical clustering of batch corrected data with Manhattan distance, with replicated samples colored. All plots represent protein-level data.

© 2021 The Authors

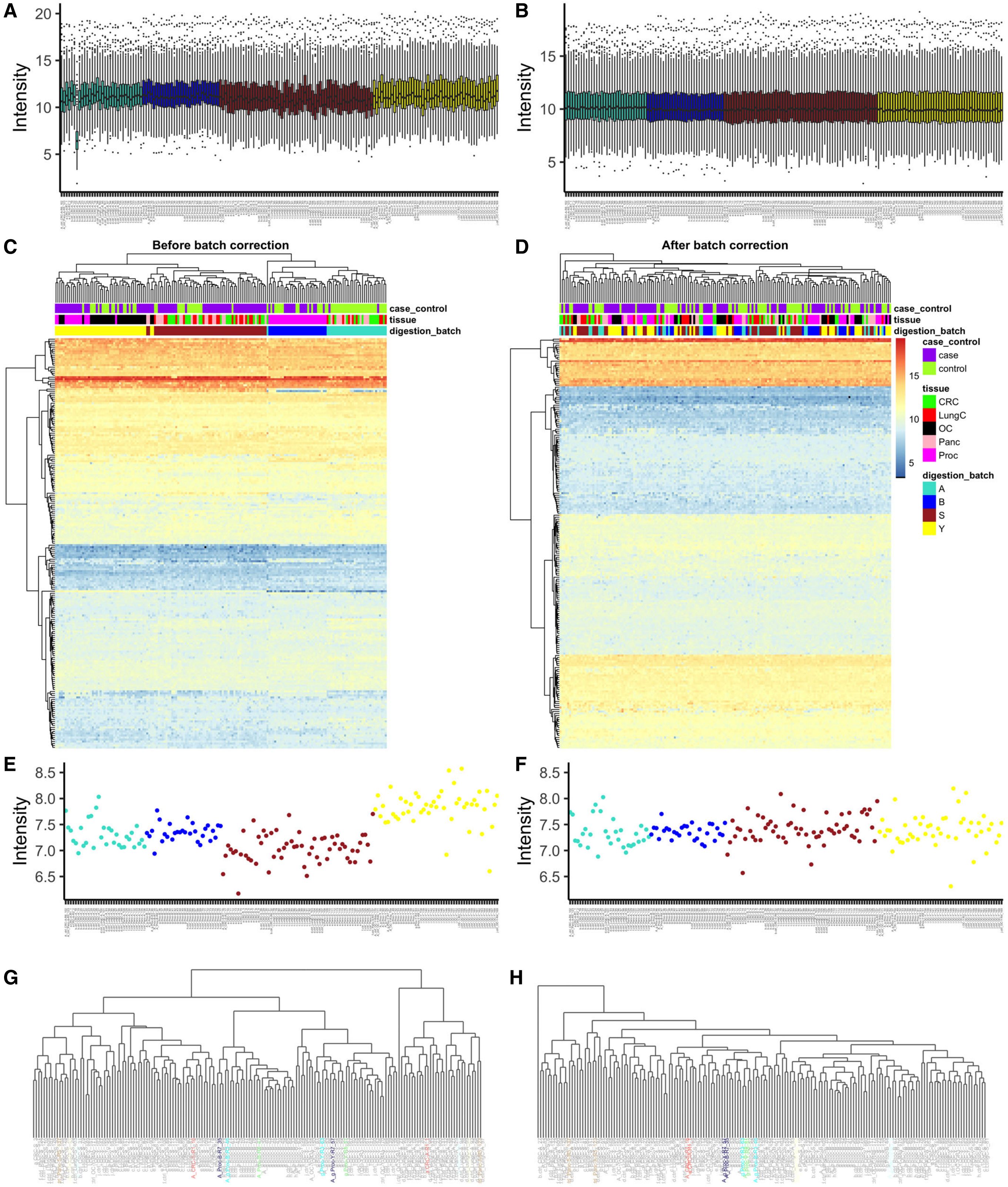

Figure EV3.

 © 2021 The Authors

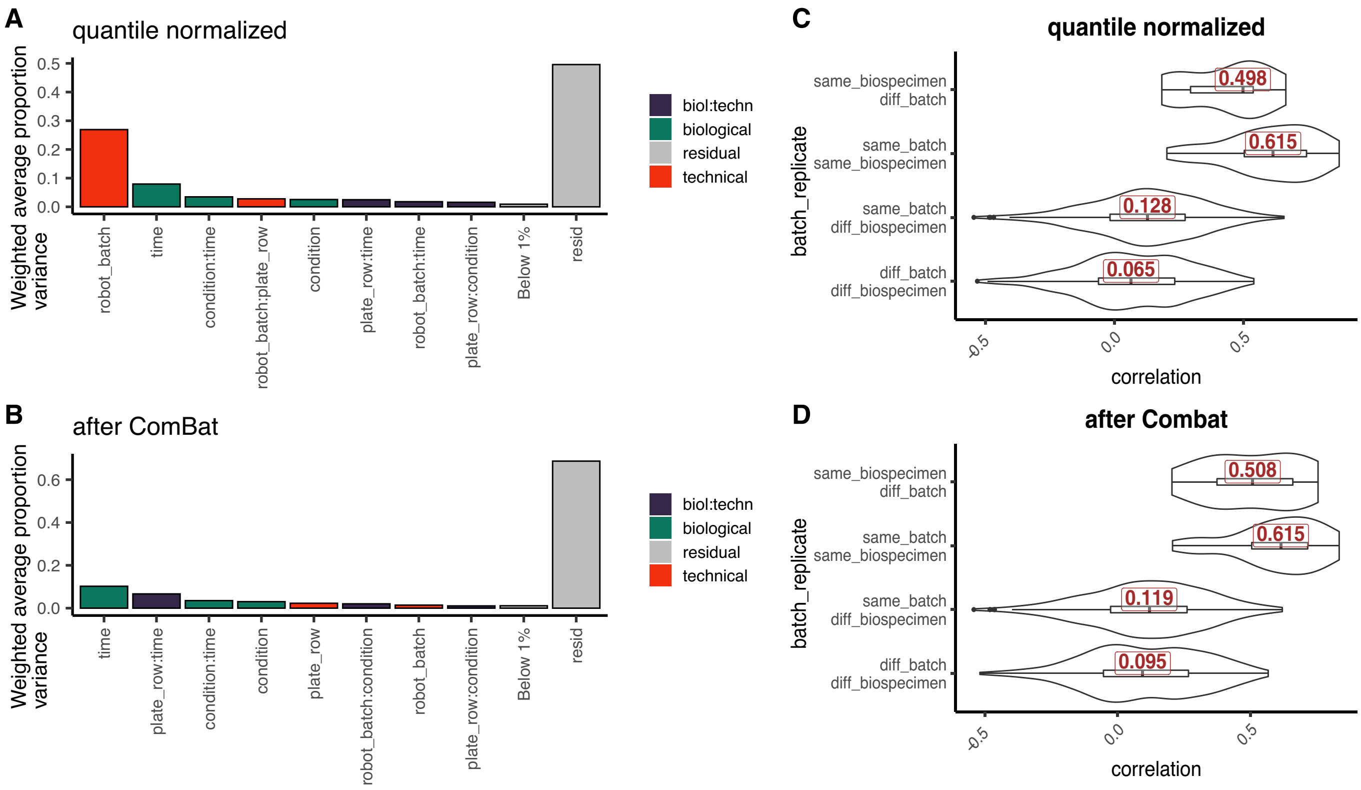

Figure EV4. Batch effects in the Bariatric surgery study.

(A) Principal Variance Component Analysis shows a clear effect of the liquid handling robot's channels (robot_batch (plate rows A and B vs. C–F)). (B) This technical factor is efficiently removed after ComBat batch correction. (C) Distribution of correlations between and within batches for different time points from the same animal and different animals prior to and (D) post-batch correction. Peptide intensities in C and D are normalized to pre-surgery intensity values. The procedure improves the correlation of samples coming from the same animal at different time points which can be used as pseudo-biological replicates in this study.

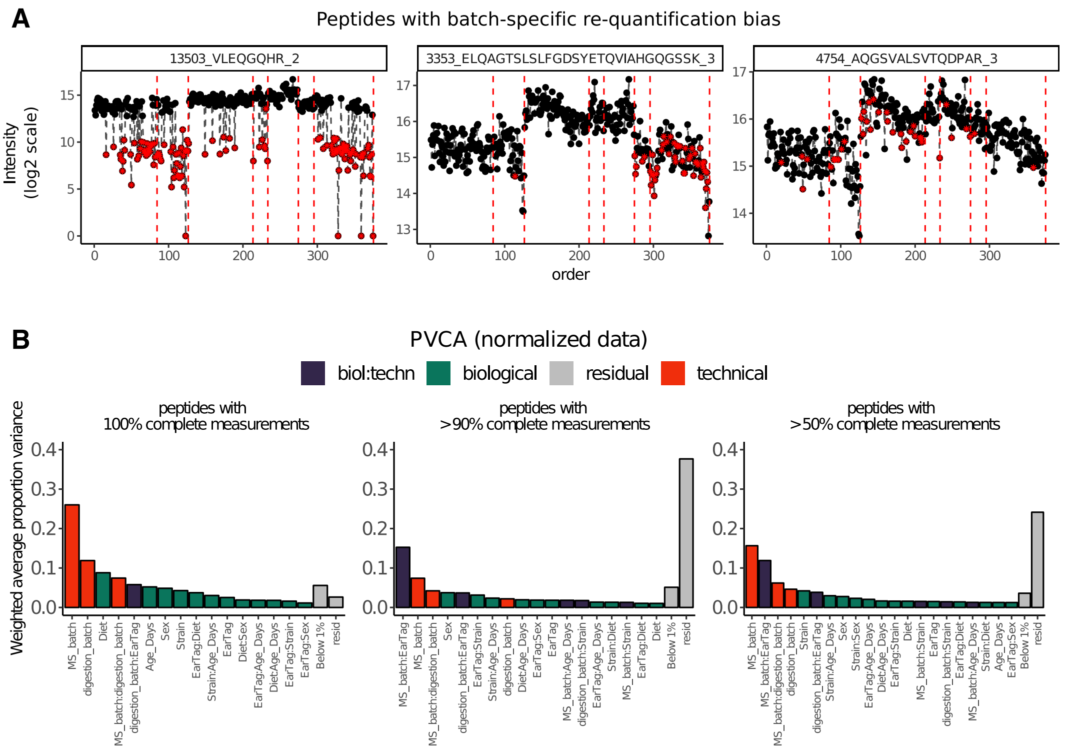

Figure EV5. Missing values and batch effects in the Aging mouse study.

(A) Re-quantification of elution traces can pick up batch-specific noise that can be drastically different (left panel) or indistinguishable (middle and right panel) from confidently identified peptide fragments, meaning that values inferred should be treated with extreme caution. Black points are regular quantifications, red points are re-quantifications; (B) PVCA can only be applied to complete matrices, and thus, missing values need to be inferred. This makes this method highly sensitive to missing values inference (here filled with 0). Depending on the completeness cutoff, variance distribution across technical and biological factors varies substantially. Note that when peptides with missing values are used in the analysis (panels center and right), a substantial portion of variance is attributed to "resid" (residual), meaning that this variance cannot be associated with any of known factors, indicating that missing value distribution is at least partially random.

 © 2021 The Authors

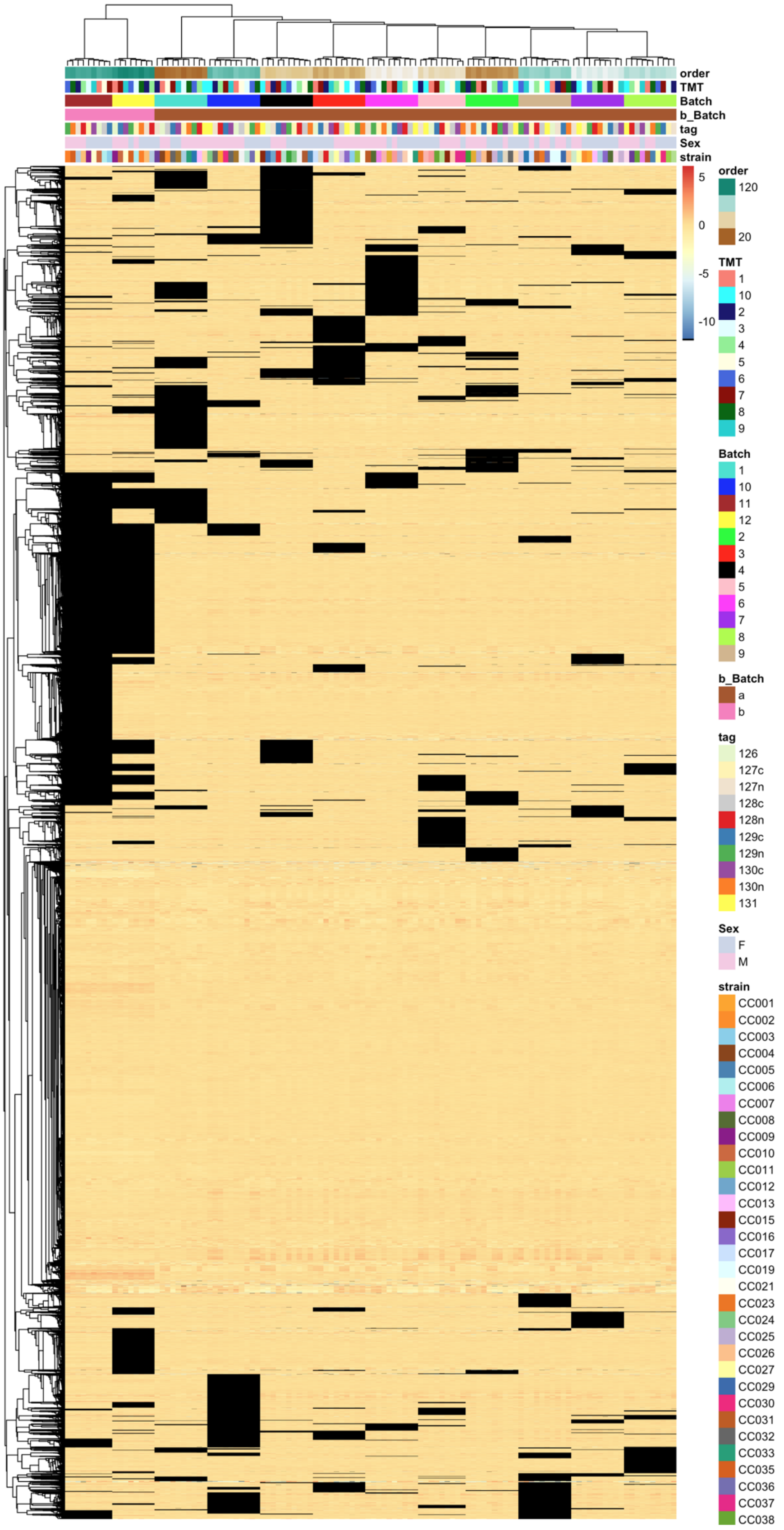

Figure EV6. Missing values in the TMT mouse study.

Heatmap of peptides with up to 30% missing values, filled with minimal values (black) shows that the missing values are primarily associated with TMT batch and this drives the clustering; compare to complete data matrix in Fig 4 in which batches 3 and 4 cluster together.

© 2021 The Authors
